# A Set of Progressive Needs

**Published:** 1919-10-25

**Authors:** 


### THE OUTLOOK AT LEICESTER INFIRMARY.

### A Set of Progressive Needs.

Mr. T. Fielding Johnson, junior, J.P., presided over the quarterly meeting of the governors of the Leicester Royal Infirmary. The meeting was notable for the outlook which it gave to the needs of progressive institutions during the next few years.

The work accomplished showed a large increase on that of the preceding period, 3,332 in-patients having been received as compared with 3,151. The attendances in the out-patient department, as, indeed, in all departments of the institution, showed that full use was being made of the institution; indeed, in the out-patient department it was felt that it was very necessary to restrict the work there to its original and proper purpose—namely, the consultative treatment of patients. In that department there was undoubtedly a fair percentage of insured patients who were entitled to receive medical treatment, as well as drugs, dressings, etc., under the National Insurance Act. Also, there were in attendance patients who for various reasons could not receive treatment from other sources. The department was to an extent undertaking the treatment of cases which local departments had not undertaken. A wasteful system was therefore in operation which should be discontinued, and which emphasised the need of co-ordination among all undertaking public health work.

The new Venereal Diseases Department was rapidly growing, they were about to introduce four male clinics per week, in addition to three female. It was very difficult to prevent overlapping among the various departments with the new service, but a recommendation had been made by the local authorities to the necessary Government Department that an entirely new department should be at once erected for the provision of separate accommodation, both for in- and out-patients. Any delay which might arise would not rest with the infirmary.

One of the most satisfactory features in the matter of income had been the amounts received from donations, which showed an increase of £1,100 on last year. The combined efforts made in town and county by means of flower shows, fêtes, parades, and the like, had been responsible for considerable sums. In connection with the Saturday Hospital Society, the workpeople had been asked to consider the increase of their contributions from 1d. to 2d. per week, with the result that a considerable increase of income at the end of the year was assured, and a much larger increase next year. The Society had long felt the need of a children's home, and active steps were being taken to provide it.

In connection with Hospital Sunday, the churches were reminded of the changed value of money, and asked, therefore, to increase their contributions.

The total expenditure to date was £1,090 more than in 1918—viz., £35,192.

### New Departments.

The new Out Patient Orthopædic Department (to the extent of £5,000 provided by the generosity of the Freemasons of Leicester) will be opened quite early in the new year.

The new Pathological Department being erected at a cost of £10,000 will also be available early next year. The governors, however, it is felt, would be wise to regard the provision of these new departments as an instalment only of a scheme which must receive consideration.

### The Housing Problem and Hospitals.

There was no newspaper that did not make reference to the housing problem. The need for housing accommodation would not be met in any locality without at once drawing attention to the necessity of increasing hospital accommodation. The re-presentation of the appeal for the Sir Edward Wood Memorial Wing, which would provide additional beds, should, therefore, not now be delayed.

### The Central Need.

The recent scourge of influenza drew attention to the urgent need for an isolation hospital, the provision of which the honorary medical and surgical staff were entirely in favour. It was thought that this building might be incorporated with a new In-Patient Orthopædic Department. Plans had accordingly been prepared for such a building, which is expected to cost £25,000, and it was hoped shortly to proceed with this important addition. No further enlargement of the work could, however, take place without the enlargement of the Nurses' Home, and no new department could be introduced without trained nurses. A new need, therefore, arose out of the provision for other needs, and if each citizen could realise that the infirmary would be benefited by his personal service there was no doubt that future demands, at present appearing a little ominous, would be met in the same way as other demands had been in past years.

The report having been adopted, cordial thanks were accorded to Dr. F. W. Bennett, Ear and Throat Surgeon, on his retirement for his valuable work for a period of nearly thirty years. A successor in the person of Dr. A. L. McLeod, who had acted for Dr. Bennett during the period of the war, had been appointed.

